# Obesity and the Genome: Emerging Insights from Studies in 2024 and 2025

**DOI:** 10.3390/genes16091015

**Published:** 2025-08-27

**Authors:** Lindsey G. Yoo, Courtney L. Bordelon, David Mendoza, Jacqueline M. Stephens

**Affiliations:** 1 Adipocyte Biology Laboratory, Pennington Biomedical, Baton Rouge, LA 70808, USA; lking51@lsu.edu (L.G.Y.); cbord66@lsu.edu (C.L.B.); dmend13@lsu.edu (D.M.); 2 Department of Biological Sciences, Louisiana State University, Baton Rouge, LA 70803, USA

**Keywords:** obesity, GWAS, incretins, polygenic risk scores, rare variants

## Abstract

Obesity is an epidemic that currently impacts many nations. The persistence of this disease is shaped by both genetic and epigenetic factors that extend beyond calorie balance. Research in the past year has revealed that epigenetic and cellular memory within adipose tissue can predispose individuals to weight regain after initial fat loss, as shown by studies indicating persistent transcriptional and chromatin changes even after fat mass reduction. Independent studies also demonstrate long-lasting metabolic shifts, such as those triggered by glucose-dependent insulinotropic polypeptide receptor (GIPR)-induced thermogenesis and sarcolipin (SLN) stabilization that also support a form of “metabolic memory” that is associated with sustained weight loss. At the neural level, rare variants in synaptic genes like *BSN* (Bassoon presynaptic cytomatrix protein), a presynaptic scaffold protein, and *APBA1* (amyloid beta precursor protein binding family A member 1), a neuronal adaptor involved in vesicular trafficking, disrupt communication in feeding circuits, elevating obesity risk and illustrating how synaptic integrity influences food intake regulation. Similarly, the spatial compartmentalization of metabolic signaling within neuronal cilia is emerging as crucial, with cilia-localized receptors G protein-coupled receptor 75 (GPR75) and G protein-coupled receptor 45 (GPR45) exerting opposing effects on energy balance and satiety. Meanwhile, genome-wide association studies (GWAS) have advanced through larger, more diverse cohorts and better integration of environmental and biological data. These studies have identified novel obesity-related loci and demonstrated the value of polygenic risk scores (PRS) in predicting treatment responses. For example, genetic variants in *GLP-1R* (glucagon-like peptide-1 receptor) and *GIPR* (glucose-dependent insulinotropic polypeptide receptor) may modulate the effectiveness of incretin-based therapies, while PRS for satiation can help match individuals to the most appropriate anti-obesity medications. This review focuses on studies in the last two years that highlight how advances in obesity genetics are driving a shift toward more personalized and mechanism-based treatment strategies.

## 1. Introduction

Obesity is a disease influenced by a variety of diverse genetic, epigenetic, neuronal, and cellular factors. While lifestyle and environmental aspects play a significant role, genetic predisposition is increasingly recognized as a foundational element, contributing substantially to an individual’s susceptibility to weight gain, the severity of obesity, and their response to interventions. Genetic factors influence how the body regulates appetite, burns energy, stores fat, and maintains metabolic health [1]. Recent studies utilizing diverse genetic approaches have provided additional insight into these complex biological pathways. For example, a canine-human genome-wide association study (GWAS) recently identified *DENND1B* (DENN domain containing 1B) as a conserved regulator of melanocortin signaling, a key neuronal pathway involved in appetite regulation and energy homeostasis [2]. Genetic variants in *DENND1B* were linked to increased adiposity in both dogs and humans. Mechanistically, *DENND1B* was shown to regulate the internalization of the melanocortin-4 receptor (MC4R), affecting downstream signaling that promotes satiety [2]. This work also highlights how genetic susceptibility interacts with environmental factors such as diet and physical activity, demonstrating that obesity risk is both complex and modifiable.

Such findings are representative of a broader wave of recent genetic discoveries, which have significantly expanded our understanding of why obesity persists and why sustained weight loss remains challenging. Genetic contributions to obesity are not determined by DNA sequence alone but involve epigenetic modifications that can reflect past metabolic states, adding a layer of complexity to understanding the genetics of obesity biology. This review will focus on recently published studies, including those from 2024 and the first half of 2025, that advance our understanding of genetic contributors to obesity. By focusing on the most recent research, this review demonstrates the dynamic nature of the genomics field, drawing particular attention to discoveries that have opened new avenues of research and therapeutic discovery. We will review recent studies on epigenetic influences on obesity and the role of new rare gene variants that influence obesity by impacting genes that influence synaptic integrity or cilia action in the brain to modulate food intake to influence obesity. We will also summarize recent GWAS studies that leverage polygenic risk scores (PRS) and underrepresented populations and reveal how incretin-based therapies show variable effectiveness influenced by genetic factors such as *GLP-1R* (glucagon-like peptide-1 receptor) and *GIPR* (glucose-dependent insulinotropic polypeptide receptor) variants. The importance of emerging obesity research is underscored by the complexity of the wide variation in obesity’s underlying causes and clinical outcomes. An example of this includes individuals with similar degrees of adiposity showing different metabolic phenotypes. Metabolically healthy obese and unhealthy obese individuals display differences in tissue remodeling, inflammatory tone, and progenitor cell dynamics, particularly within visceral fat [3]. This diversity demonstrates the challenge of treating and defining obesity and highlights the importance of integrating genetic, molecular, and therapeutic approaches to better understand its biology.

## 2. Obesity Related Genome-Wide Association Studies (GWAS)

Genome-Wide Association Studies (GWAS) have been a useful approach in human genetics research, allowing the discovery of millions of common genetic variants across the genome to identify those associated with complex traits such as obesity. In 2025, a recent preprint reported a large-scale cross-ancestry GWAS meta-analysis identified five loci that were associated with nearly a threefold increased risk of obesity: *YLPM1* (YLP Motif Containing 1), *RIF1* (Replication Timing Regulatory Factor 1), *GIGYF1* (GRB10 Interacting GYF Protein 1), *SLC5A3* (Solute Carrier Family 5 Member 3), and *GRM7* (Glutamate Metabotropic Receptor 7) [4]. These genes are involved in a range of biological processes, including RNA processing, DNA replication timing, insulin-like growth factor signaling, solute transport, and neurotransmission. Future studies will be required to determine how these genes influence obesity. Another recent preprint reported additional loci associated with severe obesity with consistent associations across diverse cohorts, including *BHLHE40-AS1* (Basic Helix-Loop-Helix Family Member E40 Antisense RNA 1), *PIWIL1* (PIWI Like RNA-Mediated Gene Silencing 1), and commonly known *PLA2R1*(Phospholipase A2 Receptor 1). Although the specific biological roles of these genes remain to be fully defined, they may participate in pathways related to adipocyte function, extracellular matrix remodeling, or circadian regulation [5].

Supporting these early discoveries, recent peer-reviewed studies have significantly advanced the field of obesity genetics and population diversity. A large-scale study, using data from up to 5.1 million people, developed new polygenic scores (PGS) for predicting body mass index (BMI) and obesity, especially in underrepresented groups [6]. These types of studies emphasize a transition in GWAS methodology toward greater ancestral diversity and improved population relevance. For example, a recent preprint study showed that ancestry-specific PRS yielded better predictive power than models based on European cohorts, showing the need for more inclusive genomic data to help achieve more impactful and equitable progress in obesity research [7]. Additionally, a study using longitudinal health records characterized the genetics of weight change across adulthood, revealing that the genetic architecture of long-term adiposity differs from that of baseline BMI. These findings reinforce the complexity of obesity genetics and the need to account for both dynamic traits and diverse populations in future research [8].

In this regard, studies like MASALA (Mediators of Atherosclerosis in South Asians Living in America) are crucial, given that South Asia is one of the most ethnically and culturally diverse regions in the world. Rather than identifying new genetic loci, the MASALA study examined whether previously identified loci from European populations could be transferable to South Asians; these included the age, number of years of US residence, adiponectin and leptin levels, employment, and marital status. Findings from the MASALA study have identified several social, demographic, and clinical factors that can serve as targets for obesity interventions among South Asians [9]. Further exploration regarding the impact of diversity in large genomic datasets included a recent two-step GWAS approach to examine the genetic architecture of obesity in Japanese and UK populations. This study was able to identify unique obesity-related genes within distinct subgroups of individuals with obesity. The findings suggest that clustering by obesity-related traits can uncover subgroup-specific genetic signals, even in smaller, more homogeneous samples [10]. Expanding the diversity of genomic data is essential for advancing obesity research in a more equitable and representative way. Future genetic studies on understudied populations have the potential to provide new and important insights into the pathogenesis of obesity.

Beyond variant discovery, GWAS studies have begun to illuminate the interaction between genetic predisposition and environmental factors. A recent study demonstrated that polygenic scores, derived from well-established obesity-associated loci such as *FTO* (Fat Mass and Obesity Associated) and MC4R, interact with lifestyle behaviors. In this analysis, individuals with high genetic risk were more susceptible to weight gain when exposed to poor dietary habits or low physical activity, whereas the same risk was lower in individuals with healthier lifestyles. Not surprisingly, these data indicated that physical activity and diet can modulate genetic obesity risk, emphasizing the dynamic nature between gene and environment interactions [11]. In addition to connecting genetic predispositions with environmental influences, GWAS have also been used to connect a patient’s genetic variation to optimize obesity treatment by developing PRS for calories to satiation (CTS_GRS_). This score moderately predicts the number of calories an individual requires before feeling full, based on genetic variation in appetite and energy regulation pathways. Subsequently, the patients’ CTS_GRS_ scores would then be used to optimize obesity treatment [12]. By utilizing GWAS, multiple advances in the scientific community have been possible. With further research, these databases may be able to identify genetic variations, environmental influences, and pharmacological interventions tailored to individual patients as we work to combat the obesity epidemic. These current findings are summarized in Table 1, which highlights key population dataset discoveries across diverse populations and their contributions to understanding obesity genetics.

## 3. Scaffold and Adaptor Genes in Presynaptic Regulation of Feeding

Building on the importance of using large datasets to identify novel obesity-related genes, understanding the mechanisms behind these genetic variants is essential for the development of more efficient therapies. Neural circuits in the hypothalamus and other brain regions continuously process internal cues related to hunger, energy levels, and reward to regulate feeding behavior. Common obesity-related variants have long highlighted brain-expressed genes that regulate appetite and satiety. Recent discoveries from rare variants, identified through a large exome-wide association study, have refined and reinforced this association. Among the most notable findings are rare protein-truncating variants in *BSN* (Bassoon presynaptic cytomatrix protein), a presynaptic scaffold gene, and *APBA1* (amyloid beta precursor protein binding family A member 1), a neuronal adaptor involved in vesicle trafficking, which are associated with large effects on adult body mass index [13]. These proteins contribute to synaptic function by organizing the presynaptic terminal and coordinating vesicle exocytosis [14,15]. The disruption of these components appears to destabilize synaptic function in circuits that regulate feeding. Variants in *BSN* were associated with markedly increased obesity risk, even surpassing that of well-known monogenic obesity genes such as *MC4R* [13]. Individuals carrying *BSN* variants had an elevated risk of type 2 diabetes and metabolic fatty liver disease but did not differ from non-carriers in childhood body size or the timing of pubertal development [13]. The impact of common genetic risk factors for obesity was stronger in individuals with *BSN* variants, suggesting that these rare mutations may intensify the effects of broader genetic susceptibility. Experiments in human stem cell-derived hypothalamic neurons indicated that partial loss of *BSN* reduced the expression of genes essential for synaptic activity, energy metabolism, and brain development. Among the affected genes were *APOE* (apolipoprotein E), *SEMA3C* (semaphorin 3C), and *NTNG1* (netrin G1), which have established roles in energy regulation and neurodegenerative disease [13].

The study also identified rare protein-truncating variants in *APBA1*, which codes for a presynaptic adaptor protein involved in vesicle trafficking, that were linked to elevated adult BMI in the discovery cohort [13,14]. *APBA1* encodes a protein that helps organize the presynaptic terminal by linking vesicles to the release machinery and maintaining stable communication between neurons [14]. Although *APBA1* has been less extensively studied than *BSN*, its involvement further supports the idea that the structural components of neurotransmission contribute to body weight regulation. These genetic studies shift the focus beyond energy sensing and metabolic outputs, highlighting the importance of synaptic integrity in determining an individual’s capacity to regulate food intake in an obesogenic environment. When the neural machinery that enables communication between neurons is disrupted, responses to satiety cues may be weakened or distorted. This raises important questions about how genetic vulnerability might interact with neurodegenerative changes, synaptic aging, or environmental stressors to influence obesity risk [13].

## 4. GPCR Networks and Ciliary Signaling

The brain’s capacity to regulate energy balance depends not only on how neural circuits are wired, but also on where and how signals are processed within individual cells. In this context, primary cilia, small finger-like projections extending from the surface of hypothalamic neurons, have emerged as crucial organizers of metabolic signaling [16]. By concentrating receptors and signaling molecules, cilia enable neurons to sense and respond to metabolic signals with precise spatial and temporal control [16]. Recent genetic and functional studies have identified GPR75 (G protein-coupled receptor 75) and GPR45 (G protein-coupled receptor 45) as cilia-localized receptors with contrasting roles in energy balance regulation [17,18]. Rare loss-of-function variants in *GPR75* are associated with reduced obesity risk in humans [18]. This protective effect was replicated in mouse models with either a spontaneous missense mutation (L144P) or a complete deletion of *Gpr75* [18]. Both of these mouse models displayed resistance to diet-induced obesity, with lower body weight and fat mass that was driven by reduced food intake rather than increased energy expenditure. Endogenous GPR75 was expressed exclusively in the brain and localized specifically to neuronal cilia, which serve as specialized signaling compartments. Variants that impaired this localization, including the mouse L144P mutation and human low-BMI associated variants, disrupted the receptor’s ability to regulate feeding behavior, highlighting the importance of ciliary compartmentalization. GPR75 signals through a Gαq-dependent pathway and alters hypothalamic gene expression in response to dietary challenge, an effect that was not observed in mice lacking leptin or *Adcy3* (adenylate cyclase 3), indicating that GPR75 requires intact components of the leptin–melanocortin pathway to exert its effects on food intake. [18]

Another cilia-localized receptor, GPR45, emerged from a forward genetic screen in mice and was found to play a critical role in central appetite regulation [17]. GPR45 localizes to primary cilia of neurons in the paraventricular nucleus of the hypothalamus, a key brain region for appetite suppression. GPR45 is required for trafficking the Gαs protein, a critical component of melanocortin receptor signaling. Loss of *Gpr45* disrupted ciliary localization of Gαs, blunted local cAMP signaling, and impaired MC4R activation, leading to increased food intake and obesity [17]. These effects occurred without changes in energy expenditure or locomotor activity, suggesting a specific defect in central satiety regulation [17]. Together, the findings from GPR75 and GPR45 underscore the importance of spatial organization within neurons. Although identified through different methods and functioning via separate pathways, both genes highlight the importance of correctly localizing and compartmentalizing signaling molecules for the brain to effectively regulate feeding.

## 5. Epigenetic and Cellular Memory in Obesity Persistence

**Figure 1:**
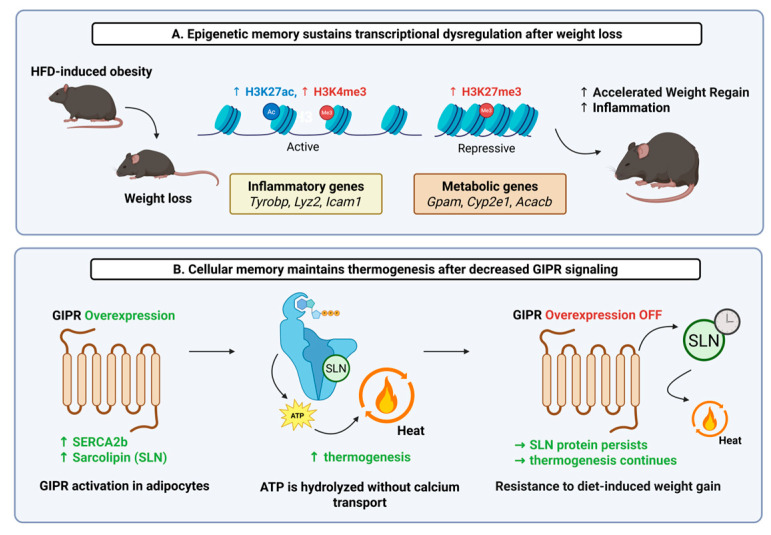
Adipose tissue memory mechanisms influencing weight regulation. (**A**) Epigenetic memory sustains transcriptional dysregulation in adipose tissue following weight loss. Obesity induces chromatin changes, including increased H3K27ac and H3K4me3 at inflammatory gene loci and increased H3K27me3 at metabolic gene loci. These epigenetic marks persist after weight loss, contributing to elevated inflammation and accelerated weight regain. (**B**) Gastric inhibitory polypeptide receptor (*Gipr*) overexpression in adipocytes drives thermogenesis via sarco/endoplasmic reticulum calcium-ATPase 2b (SERCA2b) and sarcolipin (SLN)-mediated futile calcium cycling. SLN persists even after *Gipr* overexpression is turned off, sustaining thermogenic activity and conferring resistance to diet-induced weight gain. Created in BioRender. Mendoza, D. (2025) https://BioRender.com/dyk90cx (accessed on 21 July 2025).

Extending beyond genetic variation, epigenetic modifications have emerged as powerful effectors of cellular and systemic processes. Notably, one of the most persistent challenges in obesity treatment is the high rate of weight regain following initial weight loss. This phenomenon, previously described as “obesogenic memory,” is increasingly linked to lasting biological changes that remain even after fat mass is reduced [19]. These include both long-lasting epigenetic modifications and non-genomic cellular adaptations, particularly within adipose tissue [20,21]. One newly published study has shown that adipose tissue can retain an epigenetic signature of prior obesity even after substantial weight loss. Using single-nucleus RNA sequencing in human subjects with paired multi-omic approaches in mouse models, it was revealed that adipocytes from formerly obese individuals and mice maintained transcriptional and epigenetic profiles distinct from those of never-obese controls [20]. Notably, in mouse adipocytes, histone modifications associated with active gene expression (e.g., H3K4me3/H3K27ac) remained elevated at pro-inflammatory loci, such as those for the genes *Icam1* (intercellular adhesion molecule 1), *Lyz2* (lysozyme 2), or *Tyrobp* (TYRO protein tyrosine kinase binding protein). Conversely, the repressive histone mark H3K27me3 persisted at the promoters of key adipocyte function-related genes, including *Gpam* (glycerol-3-phosphate acyltransferase, mitochondrial), *Cyp2e1* (cytochrome P450 family 2 subfamily E member 1), or *Acacb* (acetyl-CoA carboxylase beta). These changes were associated with persistent shifts in chromatin accessibility and histone modifications, which may predispose adipose tissue to a pathological response upon subsequent high-fat diet exposure (Figure 1A) [20]. Importantly, these retained molecular features in mice were associated with accelerated rebound weight gain. In addition, many of the persistent transcriptional changes were conserved between human and mouse adipocytes, supporting the translational relevance of the findings [20].

In parallel, another study has uncovered a distinct mechanism through which adipocyte function can be reprogrammed to create a “metabolic memory”. Using a mouse model with an inducible, adipocyte-specific overexpression of *Gipr*, the mouse ortholog of human GIPR, the authors showed that sustained *Gipr* induction triggered rapid and profound weight loss driven by increased thermogenesis and energy expenditure. Mechanistically, this effect was mediated by SERCA-driven futile calcium cycling, involving a shift in SERCA isoform expression and post-translational stabilization of sarcolipin. Notably, after an 8- to 12-week induction period in obese mice, the resistance to weight regain persisted even after *Gipr* transgene expression was turned off, suggesting a long-lasting shift in adipocyte metabolic function (Figure 1B). This form of “metabolic memory” reflects a durable functional adaptation that persists after the enhanced stimulus (*Gipr* signaling) is removed. Even when *Gipr* mRNA levels returned to baseline, the elevated protein levels of sarcolipin persisted. Sustained sarcolipin appears to be one player in the *Gipr*-driven metabolic memory of weight-loss maintenance [21], but it is highly likely that there are many other genetic and epigenetic factors that contribute to metabolic memory. It is important to keep in mind that durable changes in adipose tissue function are not always directly encoded in the genome. A full understanding of obesity requires considering how genetic risk interacts with epigenetic regulation, intracellular signaling, and physiological adaptation over time.

## 6. Genetic and Mechanistic Insights into Incretin-Based Obesity Therapies

In addition to gathering diverse datasets, understanding the mechanisms of genetic variations, and tracking the influence of epigenetic changes, we can also use genetic variation to inform the type of therapy chosen to treat individuals with obesity. Although initially developed for the treatment of Type 2 diabetes, incretin therapies have been FDA-approved as an obesity intervention medication due to their profound effects on weight management and satiation [22]. In light of this, key developments in obesity research have uncovered genetic factors that may influence patient responses to these incretin-based medications and the direction of future mechanistic research. One recent study explored how activating or blocking the GIPR affects body weight and food intake in male mice [23]. They discovered that GIPR agonists function independently of the glucagon-like peptide-1 receptor (GLP-1R), while GIPR antagonists require GLP-1R signaling. In addition to requiring GLP-1R signaling, GIPR antagonists activate similar genes as GLP-1R in the brain’s dorsal vagal complex, reducing the expression of genes involved in brain plasticity and synapse formation. Interestingly, GIPR antagonists still work even when *GIPR* was removed from GABA-producing and peripherin-expressing neurons, unlike GIPR agonists, which depend on GABAergic neurons [23]. While the antagonism and agonism of GIPR work through different mechanisms, the incretin medications are also mechanistically different. This is exemplified by the dual agonist drug, Tirzepatide, which mimics the actions of native GIP but shows biased signaling through the GLP-1R cAMP signaling pathway and reduced activation of the β-arrestin pathway. This biased signaling leads to decreased receptor internalization, unlike the GLP-1R monoagonist, semiglutide [24]. These recent observations underscore the complexity of understanding incretin biology. This mechanistic complexity is further compounded by the genetic variability of incretin receptors. This would include the genetic variability of the *GLP-1R* gene that may influence therapeutic response. In smaller cohort studies, several missense single-nucleotide polymorphisms (SNPs), such as *rs3765467*, *rs10305492*, and *Thr149Met*, modestly affect glycemic and gastric responses to GLP-1R agonists [25]. For example, *rs3765467* has been linked to enhanced insulin secretion and delayed gastric emptying following GLP-1 therapy [25]. However, while genetic variation in *GLP-1R* and other incretin-pathway genes can influence how patients respond to incretin-based therapies, these effects are generally small, and current evidence is insufficient to support routine genetic testing before prescribing GLP-1 therapies [26].

**Figure 2:**
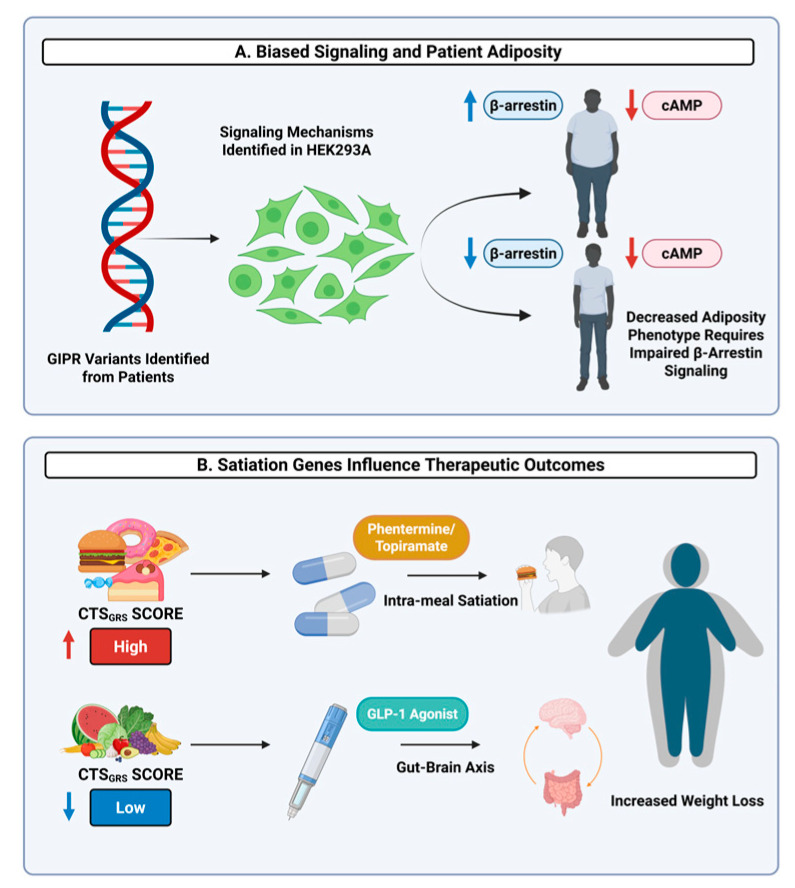
Genetic Variations Influence Patient Adiposity and Therapy Outcomes. (**A**) Naturally occurring *GIPR* variants were transfected into Human Embryonic Kidney 293A (HEK293A) cells to determine downstream signaling pathways. Variants with decreased β-arrestin recruitment and cyclic AMP signaling were associated with a leaner phenotype; however, this phenotype was not observed in variants where β-arrestin signaling was preserved. (**B**) Patients with a high CTS_GRS_ (polygenic risk score for calories to satiation) exhibited increased weight loss when treated with phentermine-topiramate compared to a glucagon-like peptide-1 (GLP-1) agonist. In contrast, patients with low CTS_GRS_ demonstrated increased weight loss when treated with a GLP-1 agonist instead of phentermine-topiramate. Created in BioRender. Mendoza, D. (2025) https://BioRender.com/sdd2jde (accessed on 21 July 2025).

Along with genetic variability, studies focusing on correlating the mechanism and therapeutic outcomes of these variabilities are also important to consider. Recent efforts have included studying naturally occurring variations in the *GIPR* gene that are associated with reduced adiposity and improved metabolic outcomes in humans [27]. These *GIPR* variants, when transfected into Human Embryonic Kidney 293A (HEK293A) cells, are characterized by an altered intracellular signaling pathway that promotes decreased amounts of β-arrestin recruitment and decreased cyclic AMP (cAMP) signaling. In comparison, participants with *GIPR* variants that lacked cAMP signaling but maintained β-arrestin recruitment did not exhibit reduced adiposity (Figure 2A) [27]. Consistent with this, *GIPR* variants with gain of function β-arrestin recruitment trended towards increased adiposity, while those with gain of function cAMP signaling trended towards decreased adiposity. Despite these positive associations with adiposity, β-arrestin knockout in HEK293A cells demonstrated impaired GIPR internalization and endosomal signaling, while knockout mice exhibited reduced glycemic control [27]. To optimize patient treatment, these genetic distinctions are critical when determining the most appropriate therapeutic medication prescribed, as metabolic responses may need different pharmacological support between the metabolically healthy and metabolically unhealthy obese individuals [3]. Consequently, these findings suggest that biased GIPR signaling may not be appropriate for future pharmacological research because while reduced β-arrestin recruitment is associated with beneficial adiposity traits, balanced β-arrestin recruitment is critical for maintaining its full metabolic function and support with glycemic control [27].

Further supporting the role of gene-phenotype interactions influencing incretin therapeutic outcomes, another study investigated whether the variability in genes associated with satiation could predict patient response to obesity medications. As detailed in an earlier section, GWAS data were leveraged to develop a polygenic risk score for calories to satiation (CTS_GRS_). This score moderately predicts the number of calories an individual needs before feeling full by utilizing the variation found in appetite and energy regulation-associated genes. In this study, individuals with high CTS_GRS_ scores responded to phentermine-topiramate treatment, a drug that enhances intra-meal satiation. Conversely, individuals with low scores responded better to liraglutide, a GLP-1 agonist that works through gut-brain axis signaling (Figure 2B). These findings suggest that polygenic profiling can help inform personalized treatment options by aligning drug mechanisms with genes associated with satiety and energy expenditure, which is particularly relevant in the context of incretin-based therapies [12].

## 7. Conclusions

Recent advances in obesity genetics have revealed a complex interplay of factors that extend beyond simple predisposition. Rather than implying a single pathway, the body of work reviewed here points to coordinated processes across multiple disciplines that shape both body weight regulation and the durability of weight control after intervention. Genome-wide studies continue to expand the map of common and rare variation while highlighting the need for broader ancestral representation and more consistent phenotyping. In parallel, mechanistic studies in brain and adipose tissue are clarifying how signals are organized within circuits and within cellular compartments, and how prior nutritional states can leave durable imprints on tissue function. Together, these findings support a model in which genetic susceptibility provides the context for interactions between neuronal control of food intake and adipose tissue programs, shaping long-term metabolic trajectories. Beyond inherited variation, obesity is also shaped by lasting biological adaptations. These adaptations are not fully reset by weight loss and can bias responses to subsequent environmental and pharmacological challenges. Persistent epigenetic features in adipose tissue provide one explanation for why the same dietary or pharmacologic intervention can produce different outcomes at different times in the same individual, while emerging circuit-level findings in the hypothalamus explain how satiety signaling can be amplified or blunted depending on synaptic organization and ciliary localization of receptors. Considering these durable tissue programs alongside neuronal mechanisms helps reconcile variability in outcomes among people who appear similar by conventional clinical measures. It also suggests that optimization of long-term care will require attention to genetic variability, tissue state, and circuit function, rather than reliance on weight alone as a proxy for risk, as obesity alone does not reliably predict metabolic health. Even among individuals with similar genetic predispositions and levels of adiposity, metabolic profiles can differ significantly.

These observations underscore the need to connect inherited risk to measurable cell states, circuit properties, behavior, and physiology, and to achieve so in cohorts that accurately reflect global diversity. Generalizability is still constrained by overrepresentation of certain ancestries, by limited resolution in the measurement of environmental and behavioral exposures, and by the fact that several mechanistic insights remain preclinical and require careful translation to humans. Predictive tools that are attractive in principle, including pathway-level genetic scores and single-variant pharmacogenetic markers, will need prospective validation and head-to-head testing within therapeutic trials before routine clinical use is justified. Progress will depend on aligning discovery with mechanisms and on designing studies that test whether biology-guided choices improve outcomes. Integrating genetic signals with single-cell, spatial, and epigenomic profiling in brain and adipose tissue can connect variants to specific cell states and circuits, while harmonized behavioral and physiological phenotyping can reveal how those mechanisms operate in daily life. Clinical trials that incorporate mechanism-anchored markers, such as quantifiable indices of satiation or pathway-level genetic scores, can directly evaluate whether treatment selection grounded in biology produces more durable weight reduction and more stable metabolic health. Clarifying when and where gut-to-brain and adipose pathways act, how those effects are maintained over time, and how they intersect with prior tissue history should enable interventions that move beyond short-term loss toward sustained benefit. As the field moves forward, integrating genomic insights with cellular and functional studies will be essential for refining obesity subtypes and improving treatment precision.

## Figures and Tables

**Table 1:** Summary of Population Studies in Obesity Research in 2024 and 2025.

Category	Findings	Gene/Loci	Implications	References
Novel Loci (2025 GWAS)	Rare variants drive severe obesity = 3x risk in carriers; five new genes	*YLPM1, RIF1, GIGYF1, SLC5A3, GRM7*	Suggests diverse biological pathways involved in obesity	[4] *
Severe Obesity Association	Multi-ancestry GWAS identified additional severe-obesity loci	*BHLHE40-AS1; PLA2R1; PIWIL1*	May relate to adipocyte function, ECM, circadian rhythm	[5] *
BMI Polygenetic Score (Multi-Ancestry)	Multi-ancestry PGS (PGSLC) improves BMI prediction across ancestries and ages	PGS_LC_ (multi-ancestry BMI PGS)	Supports early risk stratification and ancestry-inclusive scoring	[6]
Ancestry-Adjusted PRS (Indonesia)	PRS perform better in non-European populations	Ancestry-adjusted PRS (PCA)	Highlights the need for genomic diversity	[7] *
Adiposity-change GWAS (EHR)	APOE variant predicts longitudinal weight and BMI loss; replicated across cohorts	*APOE (rs429358)* + five discovery loci	Longitudinal phenotypes add discovery power; capture trajectory biology	[8]
Ancestry-Specific Insights	Clinical and social factors predict weight and waist gain in U.S. South Asians	MASALA cohort (South Asians)	Use risk profiles to target prevention	[9]
Subgroup-Specific GWAS	Phenotype clusters reveal loci - signals differ by cluster	Cluster-specific loci (Japan TMM + UK Biobank)	New methods for detecting hidden associations	[10]
Gene x Environment	Lifestyle modulates genetic risk, especially via appetite/energy pathway	*FTO*, *MC4R*	Behavior can buffer or amplify genetic susceptibility	[11]
Pharmacogenomics	Genetic scores predict drug response	CTS_GRS_	Guides treatment choice (phentermine–topiramate vs. liraglutide)	[12]

* Denotes Preprint.

## Data Availability

The original contributions presented in this review are included in the article.

## Abbreviations

The following abbreviations are used in this manuscript:

Acacb	Acetyl-CoA carboxylase beta
Adcy3	Adenylate Cyclase 3
APBA1	Amyloid Beta Precursor Protein Binding Family A Member 1
APOE	Apolipoprotein E
BHLHE40-AS1	Basic Helix-Loop-Helix Family Member E40 Antisense RNA 1
BMI	Body Mass Index
BSN	Bassoon Presynaptic Cytomatrix Protein
Cyp2e1	Cytochrome P450 family 2 subfamily E member 1
CTS_GRS_	Calories To Satiation—Genetic Risk Score
DENND1B	DENN Domain Containing 1B
FTO	Fat Mass and Obesity Associated
GABA	Gamma-Aminobutyric Acid
GIGYF1	GRB10 Interacting GYF Protein 1
GIP	Glucose-dependent Insulinotropic Polypeptide
GIPR	Glucose-dependent Insulinotropic Polypeptide Receptor
GLP-1R	Glucagon-Like Peptide-1 Receptor
Gpam	Glycerol-3-phosphate acyltransferase, mitochondrial
GPR45	G-Protein Coupled Receptor 45
GPR75	G-Protein Coupled Receptor 75
GRM7	Glutamate Metabotropic Receptor 7
GWAS	Genome-Wide Association Study/Studies
HEK293A	Human Embryonic Kidney 293A Cells
ICAM1	Intercellular Adhesion Molecule 1
Lyz2	Lysozyme 2
MC4R	Melanocortin 4 Receptor
MASALA	Mediators of Atherosclerosis in South Asians Living in America
NTNG1	Netrin G1
PIWIL1	PIWI Like RNA-Mediated Gene Silencing 1
PLA2R1	Phospholipase A2 Receptor 1
PGS	Polygenic Scores
PRS	Polygenic Risk Score
RIF1	Replication Timing Regulatory Factor 1
SEMA3C	Semaphorin 3C
SERCA	Sarcoplasmic/Endoplasmic Reticulum Calcium ATPase
SNP	Single Nucleotide Polymorphism
SLN	Sarcolipin
SLC5A3	Solute Carrier Family 5 Member 3
Tyrobp	TYRO Protein Tyrosine Kinase Binding Protein
YLPM1	YLP Motif Containing 1
